# The vestibular labyrinth is more robust than previously thought—Lessons from surgical removal of intracochlear schwannoma

**DOI:** 10.1007/s00106-022-01175-9

**Published:** 2022-06-01

**Authors:** Stefan K. Plontke, Torsten Rahne, Ian S. Curthoys, Bo Håkansson, Laura Fröhlich

**Affiliations:** 1 Department of Otorhinolaryngology, Head & Neck Surgery, Martin Luther University Halle-Wittenberg, University Medicine Halle, Ernst-Grube-Str. 40, 06120 Halle (Saale), Germany; 2 Vestibular Research Laboratory, School of Psychology, The University of Sydney, Sydney, NSW Australia; 3 Electrical Engineering, Chalmers University of Technology, Gothenburg, Sweden

The receptors for hearing and balance are housed together in the labyrinth of the inner ear and share the same fluids. It was widely believed that surgical damage to either receptor system causes certain permanent loss of the receptor function of the other. However, anecdotal reports in individual patients of at least partial preservation of cochlear function after major surgical damage to the vestibular division and vice versa have called that principle into question. Recently, we showed in a large case series that after major trauma to the cochlea for surgical removal of intracochlear schwannoma, the vestibular receptors continue to function normally. This was demonstrated by specific, objective function tests for each of the peripheral vestibular receptors before and after surgery [1].

## Subtotal cochleoectomy as an inadvertent surgical trauma model

**Fig. 1:**
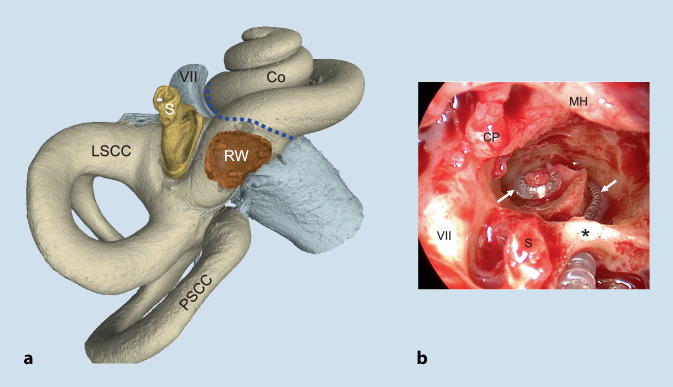
Surgical separation of the cochlear and vestibular labyrinthine parts. **a** Three-dimensional reconstruction of the human inner ear based on micro-computed tomography (µCT) images. The *dashed blue line* shows the area where the cochlear and the vestibular labyrinthine parts are surgically separated (µCT: Matthias Menzel, Fraunhofer Institute for Microstructure of Materials and Systems IMWS Halle (Saale), Germany; with permission). **b** Intraoperative endoscopic view showing a right middle ear with an opened cochlea (subtotal cochleoectomy) after removal of an intracochlear schwannoma. The electrode carrier of the cochlear implant (→) is placed around the modiolus. During surgery, the bony arch (*asterisk*) of the round window is preserved. Despite this major trauma to the cochlea, vestibular function was preserved as shown by objective receptor tests. *Co* cochlea (second turn), *CP* cochleariform process, *LSCC* lateral semicircular canal, *MH* malleus handle, *PSCC* posterior semicircular canal, *S* stapes, *VII* facial nerve (in **a** distally from geniculate ganglion not shown), *RW* round window, *asterisk* preserved round window arch

During the past few years, surgical therapy of rare intracochlear schwannomas—often presenting with symptoms of sudden hearing loss or Menière’s disease—has been shown to be an effective way of managing these tumors including successful hearing rehabilitation with cochlear implants. Regardless of the specific technique used for tumor removal, this kind of surgery causes major trauma to the cochlea, and a disruption of the membranous parts of the entire inner ear would be very likely or even expected. The technique of partial or subtotal cochleoectomy that we usually use for removal of intracochlear schwannomas has thus—inadvertently—become a surgical trauma model of the inner ear and provided new insight into the function of vestibular labyrinth sensory regions after trauma (Fig. 1).

## Vestibular function tests show preserved receptor function

As part of a standard test protocol, we performed serial, specific state-of-the-art objective vestibular function tests before and after surgical trauma (subtotal cochlear removal) for treatment of intracochlear tumors on 27 consecutive patients in our university audiology, hearing implant, and skull base center. Vestibular function was assessed by calorics (low-frequency response of the lateral semicircular canal), vestibulo-ocular reflex by video head impulse test (vHIT) of the three semicircular canals, and cervical and ocular vestibular-evoked myogenic potentials (cVEMP, saccule and oVEMP, utricle). Otolith function was measured by bone conducted vibration, which was used because of the postoperative conductive hearing loss after removal of the incus. Preoperative and postoperative distributions were compared with paired *t* tests.

The statistical analysis showed that there was no significant difference between pre- and postoperative measures for all tests of the five vestibular sensory regions. The incidence and direction of spontaneous nystagmus did not change between pre- and postoperative testing. In the majority of patients, calorics showed approximately the same or an improved low-frequency response of the lateral semicircular canal compared with the preoperative situation. Only two patients showed deteriorated function in this test. The vHIT gain was still normal after surgery in most patients. One patient improved slightly but the gain stayed at preoperatively measured abnormal values, while in two patients vHIT gain deteriorated. In the anterior and posterior planes, only three and two patients showed a deterioration to postoperative pathological gain, respectively. In both planes, two patients improved slightly (but the gain stayed abnormal) and one patient improved to postoperative normal results.

Normal otolith function of the saccule was observed postoperatively in most patients, the same being observed for utricular function.

Successful hearing rehabilitation with the cochlear implant and continuous improvement over 12 months was shown by word recognition at normal speech level (65 dB SPL, [1]).

## Hypotheses for robustness of the vestibular labyrinth

Although the mechanisms and reasons for these observations are yet unknown, there appear to be three major aspects explaining this phenomenon: anatomical, physiological, and surgical.

Firstly, the anatomy appears to be in favor of such a traumatic intervention. The small duct that links the endolymphatic spaces of the cochlea with those of the vestibular labyrinth—the ductus reuniens—is very thin (diameter < 0.2 mm in the smallest part) and is probably sealed after subtotal cochlear removal.

Secondly, it appears that sufficient endolymph-generating cells in the vestibular labyrinth keep the vestibular sensory organ functioning. Mainly these are the dark cell epithelium and subepithelial melanocytes of the vestibular division of the labyrinth, which are functionally comparable to the marginal and intermediate cells of the stria vascularis, respectively. Thus, the regulation of endolymph K^+^ homeostasis through a fine balance between secretion and absorption by epithelial cells is maintained, providing the precondition for mechano-transduction in the vestibular sensory hair cells.

Lastly, the surgical technique, characterized by extreme care, avoiding suction near the vestibule, working “under water” as often as possible, and using artificial perilymph-like irrigation solution together with careful soft tissue packing of the ductus reuniens area in the hook region of the cochlea, appears to contribute to this success.

## Practical conclusion

- The observation that vestibular function is not only able to be preserved but can most commonly be preserved following subtotal cochleoectomy challenges a common conception of the negative impact of cochlear surgery on vestibular function.
- This may effectively change future cochlear surgery indications and techniques.
- Together with the observation of above-average results in hearing rehabilitation with cochlear implantation, this strengthens the recommendation for a surgical management strategy of intracochlear schwannomas and cochlear implantation, rather than radiotherapy or a “wait & test & scan” strategy.
- The anatomical and physiological backgrounds for the robustness of the vestibular labyrinth to surgical trauma, however, need to be further explored.

## Declarations

All procedures performed in studies involving human participants or on human tissue were in accordance with the ethical standards of the institutional and/or national research committee and with the 1975 Helsinki declaration and its later amendments or comparable ethical standards. Informed consent was obtained from all individual participants included in the study.

The supplement containing this article is not sponsored by industry.
